# Effect of Repeated Remote Ischemic Preconditioning on Peripheral Arterial Disease in Patients Suffering from Intermittent Claudication

**DOI:** 10.1155/2019/9592378

**Published:** 2019-12-10

**Authors:** Mehmet Balin, Tarık Kıvrak

**Affiliations:** Department of Cardiology, Firat University, School of Medicine Hospital, Elazığ, Turkey

## Abstract

**Background/Objective:**

Intermittent claudication (IC) is the symptom of peripheral artery disease (PAD) and causes functional disability. Remote ischemic preconditioning (RIPC), is a phenomenon in which a short period of sub-critical ischemia, protects tissues against ischemia/reperfusion/injury. We considered to test the hypothesis that RIPC in PAD patients suffering from IC would increase muscle resistance to ischemia and thus improve walking-capacity.

**Materials/Methods:**

A total of 63 patients with proven-IC underwent two treadmill tests (graded treadmill protocol) with a 28-day interval in between. Patients were consecutively assigned for the non/RIPC-group and RIPC-group procedure one by one. Patients received 5-cycles of alternating 5-minute inflation and 5-minute deflation of blood-pressure cuffs on nondominant upper-limb every day for four weeks. Initial claudication distance (ICD), total walking distance (TWD) and time to relief of claudication (TRC) were recorded during procedure.

**Results:**

Patients receiving-RIPC exhibited a marked increase in ICD and TWD between basal and last tests: 209.1 ± 15.4 m vs. 226 ± 15.0 m and 368.8 ± 21.0 m vs. 394 ± 19.9 m, respectively (*p* < 0.001). In addition, patients receiving-RIPC represented a significant decrease in TRC between basal and last tests: 7.8 ± 1.3 min vs. 6.4 ± 1.1 min, respectively (*p* < 0.001). Patients not receiving-RIPC did not exhibit improvement in ICD, TWD, and TRC between basal and last tests: 205.2 ± 12.1 min vs. 207.4 ± 9.9 min, 366.5 ± 24.2 min vs. 369.4 ± 23.2 min and 7.9 ± 1.4 min vs. 7.7 ± 1.3 min, respectively (*p* > 0.05).

**Conclusion:**

A significant increase in ICD and TWD were observed in last/treadmill test in RIPC-group. In addition, a significant decrease in TRC was observed in last/treadmill test in RIPC-group. In non/RIPC-group, no improvement was observed in ICD, TWD and TRC.

## 1. Introduction

Peripheral arterial disease (PAD) is a manifestation of systemic atherosclerosis that affects adults >40 years of age and causes an increased risk for cardiovascular morbidity and mortality [1–5]. Intermittent claudication (IC) is the classical symptomatic clinical presentation of PAD, and is associated with reduced exercise capacity and poor quality of life among these patients [1, 6, 7]. Conventional medical therapies to reduce the burden of lower extremity symptoms in patients with PAD are limited. Revascularization by endovascular interventional or/and surgical reconstruction is used to treat lifestyle-limiting claudication if patients do not respond adequately to conventional medical therapy. Indeed, lower extremity revascularization with endovascular approaches has undergone a dramatic increase in use. Though endovascular therapy improves blood flow and function, there are significant associated risks, and durability may be limited, especially in infra-inguinal disease.

Ischemic preconditioning is an endogenous mechanism of protection whereby short periods of sub-critical ischemia performed in an organ confer protection against further ischemia in that same organ [8]. Ischemic preconditioning was first described in 1986 by Murry et al. [9] as an increase in cellular resistance to myocardial ischemia when the heart is exposed to periods of brief nonlethal ischemia interspersed with reperfusion. In 1993, Pryzklenk et al. [10] demonstrated that an increase in cell resistance to ischemia also occurred in other tissues that were not directly subjected to ischemia. This phenomenon was named remote ischemic preconditioning (RIPC) [11–13]. Saes et al. [14] showed that short-term RIPC application increased the initial claudication distance in PAD patients with IC; nevertheless, RIPC did not affect the total walking distance of the study patients.

Based on current evidence of RIPC occurring in other tissues, this study was designed to determine whether relative long-term RIPC induced by transient arm ischemia had a beneficial effect on the initial claudication distance (ICD), the total walking distance (TWD) and time to relief of claudication (TRC) in PAD patients with intermittent claudication.

## 2. Methods

This study was performed at the Department of Cardiology, School of Medicine Hospital, Firat University after receiving approval from the local ethics committee at University of Firat, Faculty of Medicine and conducted in accordance with the Declaration of Helsinki. The patients who were medically treated with the diagnosis of PAD and who had IC in the cardiology outpatient clinic of Firat University Medical Faculty Hospital were recruited by phone. Demographic information, height, weight, cardiovascular risk factors, comorbid conditions, a history of claudication, ankle-to-brachial index and a list of current medications were recorded during the medical history interview. All of the participants signed informed consent forms prior to their enrollment. All patients who were recruited by phone were screened. To be eligible, patients had to exhibit an ankle-to-brachial index <0.90 either in the symptomatic leg (Rutherford Classification Stage-I/Category-1 [15], each patient had documented by multislice computed tomography unilateral or bilateral infra-inguinal disease with significant stenosis present in one or more arteries, or in both, as well as scoring positive on a treadmill test performed in our laboratory. An abnormal test was defined as the combination of claudication experienced during the treadmill test, ICD > 150 and TWD < 750 m. Patients having the following criteria were excluded from the study: stable/unstable angina pectoris; myocardial infarction in the last three months; ejection fraction ≤30%; orthopedic disability and humeral systolic blood pressure at rest >200 mmHg.

After the baseline measurements, patients were randomized into RIPC-group and non/RIPC-group. The patients were given sufficient and detailed information about graded treadmill protocol testing, RIPC and non/RIPC protocols before the study. All patients were familiar with the graded treadmill protocol test, RIPC and non/RIPC protocols. Both groups of patients underwent graded treadmill protocol testing before the RIPC and non/RIPC protocol, and then RIPC-group was tested again on the same treadmill protocol after receiving daily RIPC for four weeks. Non/RIPC-group was tested again on the same treadmill protocol after receiving daily non/RIPC for four weeks. Patients performed a progressive, graded treadmill protocol (2 mph, 0% grade with 2% increase every 2 minutes) until maximal claudication pain as previously described [16]. The ICD (which describes the maximum distance a patient can walk without experiencing leg pain), the TWD (which refers to the distance walked before the patient could not continue walking) and the TRC (which describes as the period in which leg pain and calf stiffness completely resolved) were recorded in each test. The exercise was discontinued at the patient's request. The participants were advised to avoid consuming the following substances during the medical history interview, which have been suggested to interfere with the process of RIPC, within four hours of the test: cilostazol, sildenafil, dipyridamole, glibenclamide, aminophylline, nicorandil, phenylephrine, angiotensin-converting enzyme inhibitors, angiotensin receptor blocker II, statins and steroids, caffeine and alcohol.

RIPC was applied by modifying a previously described protocol [17], which is described in detail below. In both the RIPC-group and non/RIPC-group procedures, an inflated cuff was positioned on the nondominant upper limb of the participant 5 times for 5 minutes each time. Between each period of inflation, the cuff was deflated for 5 minutes. Both RIPC and non/RIPC protocols were performed daily at the same time of day and for four weeks at home by patients. Both RIPC and non/RIPC protocols were completed in a total of 50 minutes. The blood-pressure cuffs were inflated to a pressure of 200 mmHg and 10 mmHg in the RIPC-group and non/RIPC-group procedures, respectively. Participants were called by phone twice a week and received information about the RIPC and the non/RIPC protocol.

### 2.1. Sample Size and Statistical Analysis

Sample size calculation was performed using the Number Cruncher Statistical System (NCSS)–Power Analysis and Sample Size (PASS) software. Based on the differences in the mean scores of the QoL and OA scales obtained from the patients in the pilot study, the alpha level was set at 0.05 and power as 80%. Thereby, minimum 30 patients were included in each group.

Statistical analyses were performed using SPSS 22.0 statistical package for Windows. Kolmogorov–Smirnov test assessed normal distribution. Continuous data were expressed as a mean ± standard deviation, while categorical data presented as percentages or number of patients. Chi square test was used for comparison of categorical variables, while student *t*-test or Mann–Whitney *U* test was used to compare parametric and nonparametric continuous variables, respectively. The effect of RIPC and non/RIPC protocols in each groups were assessed by two-way analysis of variance (ANOVA) [time X group] for repeated measures. When significance was obtained, Newman–Keuls post hoc test was used to identify the differences. A *p*-value of <0.05 was considered statistically significant.

## 3. Results

In this study, 63 patients (%73 males and %27 females) were analyzed. Mean age, duration of disease, body mass index and ankle-to-brachial index of all patients were 63.0 ± 8.2 years, 42.1 ± 14.5 months, 24.6 ± 2.6 kg/m^2^and 0.63 ± 0.1, respectively. Physical examinations of the upper limbs revealed normal physiology in all the participants. The baseline characteristics of the both group patients are shown in Table 1. No differences were observed between the groups regarding age, gender, body mass index, ankle-to-brachial index, hypertension prevalence, diabetes mellitus prevalence, dyslipidemia prevalence, myocardial revascularization (percutaneous and surgical) history, myocardial infarction history, carotid artery atherosclerosis, stroke, current smoker and medication. The treadmill test parameters within the group and between the groups were summarized in Tables 2 and 3, respectively. In the analysis based on two-way analysis of variance (ANOVA) [time X group] for repeated measurements: *P*-value of group, time and interaction were 0.036, <0.001, <0.001, respectively. In addition, improvement in exercise parameters were more evident in nondiabetic patients in the RİCP-group, but this improvement did not reach statistical significance. This could be more evident if the patient volume was greater. Patients not receiving RIPC did not exhibit improvement in ICD, TWD and TRC between the basal and last tests. In addition, when the last exercise tests were undergoing in RİCP-group patients, in 25.8% of the patients, we firstly observed an increase in the claudication, then a partial decrease and finally a re-increase.

## 4. Discussion

Intermittent claudication is the classic symptomatic form of PAD, affecting millions of people worldwide and is increasing rapidly with the aging world population [1]. Patients with claudication experience significant functional disability resulting in a sedentary lifestyle and reduced quality of life [18–20]. Regarding symptomatic PAD, different therapeutic strategies are available: risk factors modification, exercise therapy, pharmacotherapy and surgical/interventional revascularization [21, 22]. Despite all these treatment modalities, satisfactory results have not yet been reached in the treatment of IC in PAD. Research on new drugs and new methods is currently underway. Our study is a research conducted within this scope.

Przyklenk et al. [10] demonstrated that brief cycles of ischemia/reperfusion of the circumflex coronary artery protected remote virgin myocardium from the left anterior descending coronary artery occlusion. The phenomenon of RIPC has been described in different organs and tissues, emerging as a strategy of inter-organ protection against the effects of acute ischemia/reperfusion injury. Kerendi et al. [23] demonstrated that brief ischemia/reperfusion applied to a distant organ at the onset of myocardial reperfusion reduces myocardial infarct size. The preconditioning stimulus starts in the remote organ or tissue to reach the target organ by different pathways. The involvement of a neurogenic pathway in remote cardio-protection has been demonstrated by different authors [24, 25]. Takaoka et al. [26] reported that plasma adenosine concentrations in the carotid artery were elevated following renal ischemia and reperfusion in a rabbit model of remote preconditioning by renal artery occlusion. Currently, it is believed that transmission of the RIPC signal to the target organ is multifactorial, requiring a combination of humoral, neuronal, and systemic mechanisms, and may be model-dependent. Indeed, the release of humoral factors in response to RIPC is dependent on sensory innervation to the preconditioned limb [27]. Gedik et al. [28] showed that the transfer of RIPC pig plasma attenuated ischemia/reperfusion induced mitochondrial reactive oxygen species production after improved adenosine diphosphate-stimulated complex I respiration, indicating that mitochondria are involved in reducing ROS formation, supporting the notion that mitochondria are a target organelle of the protection provided by RIPC. Of the few RIPC studies that have been conducted in human subjects, some have shown that RIPC protects endothelial function after ischemia-reperfusion injury [17, 29, 30], and other studies have shown the protection of myocardial cells in patients undergoing cardiac operations [31, 32].

RIPC is currently of particular interest in PAD patients with IC. RIPC may at least partially explain the success of physical training in the treatment of patients with IC. The mechanism by which exercise improves vascular mediated IC symptoms is poorly understood. Moreover, the first concept of physical activity that promotes increased collateral circulation by stimulating neo-angiogenesis does not have clear evidence [33]. Current theories have aimed to link this development to changes in exercise-induced muscle metabolism at walking distance [34]. Repeated ischemia-reperfusion events caused by physical training may be stimulating for intracellular biochemical changes leading to more efficient use of oxygen by the muscle and improvement of endothelial function. This theory is based on the RIPC concept. Improving quality of life by increasing ICD and TWD in PAD patients with IC is the main treatment goal.

In this study, we compared the ICD, TWD, and TRC receiving and not receiving RIPC in each treadmill test. A significant increase in the ICD and TWD were observed in the last treadmill tests in RIPC-group, and this increase was greater in patients who had got diabetes mellitus. These findings suggested that repeated RIPC contributed to an increase in ICD and TWD. Thus, RIPC-group averaged a significantly higher ICD and TWD in the last treadmill test when compared to the basal treadmill test. In addition, a significant decrease in the TRC was observed in the last treadmill tests in RIPC-group, this decrease was less in patients who had diabetes mellitus. In addition to all these, when the last exercise tests were being undergone in RİCP-group patients, in 25.8% of the patients, we firstly observed an increase in the claudication, then a partial decrease and finally a re-increase. This observation was interpreted as a warm-up phenomenon. None of the patients with warm-up phenomenon had a history of diabetes mellitus.

In the non/RIPC-group, no improvement was observed in ICD, TWD and TRC parameters. When the last treadmill tests of the RIPC- group and non/RIPC-group were also compared, it was observed that the ICD, TWD and TRC parameters of the RIPC- group were significantly improved. In the studies carried out within the framework of this concept; Saes et al. [14] showed that short-term RIPC application increased the initial claudication distance in PAD patients with IC; nevertheless, RIPC did not affect the TWD of these patients. Delagarde et al. [35] was demonstrated that RIPC did not improve walking distance in PAD patients with IC. These contradictory results can be explained by the cycle and duration of the RIPC application. In our study, the cycle of RIPC was more (five cycles) and the duration of RIPC was longer (four weeks).

### 4.1. Study Limitations

The major limitation of this work is the small sample size of participants. The patients were not completely blinded to the procedure. In addition, we only tested one RIPC protocol, investigated the four-weeks phase of the preconditioning.

## 5. Conclusions

Among PAD patients suffering from IC; patients receiving RIPC were showed an improvement of ICD, TWD, and TRC parameters compared to patients not receiving RIPC. A significant increase in the ICD and TWD were observed in the last treadmill tests in RIPC-group, and this increase was greater in patients who had no diabetes mellitus. In addition, a significant decrease in the TRC was observed in the last treadmill tests in RIPC-group, this decrease was less in patients who had diabetes mellitus. Future large volume studies are needed to confirm the effect of this procedure in these patients. If this procedure efficacy is confirmed with large volume patients' studies, this method may be a light of hope for these patient's treatment in the future.

## Figures and Tables

**Table 1:** General characteristics of patients with intermittent claudication.

	RIPC-group (*n* = 31)	Non/RIPC-group (*n* = 32)	*p*-value
Age (years)	63.5 ± 9.0	62.2 ± 7.5	0.27
Male gender (%)	77.4	68.8	0.31
PAD evolution (months)	42.2 ± 14.1	41.6 ± 14.9	0.83
Body mass index (kg/m^2^)	25.9 ± 2.3	24.6 ± 2.6	0.98
Ankle-brachial index	0.62 ± 0.1	0.63 ± 0.1	0.87
Hypertension (%)	61.3	62.5	>0.05
Diabetes mellitus (%)	35.5	34.4	>0.05
Dyslipidemia (%)	36.5	37.5	>0.05
Current smoker (%)	61.9	62.5	>0.05
Revascularization history (%)	22.2	21.9	>0.05
Atherosclerosis CAD (%)	15.9	15.6	>0.05
Aspirin (%)	71.4	65.6	>0.05
P2Y12 inhibitors (%)	54.8	53.1	>0.05
Statins (%)	49.2	50.0	>0.05
Beta-blockers (%)	28.6	28.1	>0.05
Antihypertensive drugs (%)	58.7	59.4	>0.05
Insulin (%)	28.6	28.1	>0.05
Cilostazol (%)	17.5	18.8	>0.05
Distribution of the peripheral arterial lesions (%)			>0.05
SFA	41.9	40.6	
Popliteal artery	9.6	12.5	
ATA	54.8	50.0	
PA	25.8	21.8	
PTA	51.6	43.7	
Presence of bilateral lesion (%)	45.1	46.8	>0.05

Data are expressed as mean ± standard deviation, number (%) or median (25th–75th percentile). PAD: Peripheral artery disease; CAD: Carotid artery disease; SFA: Superficial femoral artery; ATA: Anterior tibial artery; PA: Peroneal artery; PTA: Posterior tibial artery.

**Table 2:** Treadmill test parameters within groups.

	RIPC-group (*n* = 31)	*p*-value	Non/RIPC-group (*n* = 32)	*p*-value
ICD (m): basal vs. last test	209.1 ± 15.4 vs. 226 ± 15.0	<0.001	205.2 ± 12.1 vs. 207.4 ± 9.9	0.13
TWD (m): basal vs. last test	368.8 ± 21.0 vs. 394 ± 19.9	<0.001	366.5 ± 24.2 vs. 369.4 ± 23.2	0.21
TRC (min): basal vs. Last test	7.8 ± 1.3 vs. 6.4 ± 1.1	<0.001	7.9 ± 1.4 vs. 7.7 ± 1.3	0.11

Values are expressed as the mean ± standard deviation. ICD: Initial claudication distance, TWD: Total walking distance; TRC: Time to relief of claudication.

**Table 3:** Treadmill test parameters between groups.

	RIPC-group (*n* = 31)	Non/RIPC-group (*n* = 32)	*p*-value
ICD (m): basal test	209.1 ± 15.4	205.2 ± 12.1	0.27
ICD (m): last test	226.6 ± 15.0	207.4 ± 9.9	<0.001
TWD (m): basal test	368.8 ± 21.0	366.2 ± 24.2	0.70
TWD (m): last test	394.8 ± 19.9	369.4 ± 23.2	<0.001
TRC (min): basal test	7.8 ± 1.3	7.9 ± 1.4	0.85
TRC (min): last test	6.4 ± 1.1	7.7 ± 1.3	<0.001

Values are expressed as the mean ± standard deviation. ICD: Initial claudication distance, TWD: Total walking distance; TRC: Time to relief of claudication.

## Data Availability

The data used to support the findings of this study are included within the article.
